# Multidimensional poverty among persons with disabilities in Colombia: Inequalities in the distribution of deprivations at the municipality level

**DOI:** 10.1371/journal.pone.0286983

**Published:** 2023-06-29

**Authors:** Mónica Pinilla-Roncancio, Gustavo Cedeño-Ocampo

**Affiliations:** Centre of Sustainable Development Goals (CODS), School of Medicine, Universidad de los Andes, Bogotá, Colombia; Australian National University, AUSTRALIA

## Abstract

According to the national population Census of Colombia, 4.1 per cent of the population lives with a disability. Although information is available on the number of persons with disabilities in the country, little information exists on their levels of multidimensional poverty and deprivation, especially at the province of local level. Aiming to contribute to the analysis of the levels of poverty of persons with disabilities living at the municipal/province level, this study computes and analyses the levels of multidimensional poverty in households with and without members with disabilities in the 1.101 municipalities of Colombia. Using the 2018 national population census, we computed the percentage of people living with disabilities in each of the municipalities of the country, then we computed their levels of poverty and deprivation and analysed the difference between households with and without members with disabilities. We also analysed the availability of teachers and schools providing services for children living with disabilities and deprivations in terms of school attendance. The results reveal that households with members with disabilities are poorer than households without members with disabilities, These households present higher deprivations according to most indicators, and the intensity of their poverty is higher. In addition, households with members with disabilities usually present higher levels of deprivation in school attendance and live in municipalities where there is no provision of inclusive schools. These results highlight the importance of implementing specific policies designed to reduce the levels of poverty of persons with disabilities and their families and to guarantee their access to basic opportunities and services.

## 1 Introduction

Around 16 per cent of the global population lives with some form of disability [1], and it is expected that this percentage increases in low- and middle-income countries (LMICs), especially countries that have experienced violent conflict, such as Colombia. According to the national household and population census of Colombia, approximately 4.5 per cent of the population older than the age of five lives with a disability, and visual impairments are the most prevalent [2].

In recent decades the analysis of the levels of income and multidimensional poverty of persons with disabilities has increased in LMICs [3]. Indeed, a systematic review conducted in 2017 found that 81 per cent of the studies provided evidence of a positive association between disability and poverty. Contrary to expectations, the number of studies that presented a positive association between both conditions increased with the level of income of the country in question [3]. This might be the result of the disability development gap, which implies that persons with disabilities are usually left behind by development policies [4]. In addition, when the levels of multidimensional poverty for persons with disabilities are analysed, the results reveal that these individuals are more likely to be multidimensionally poor and have on average a larger number of deprivations [5–8]. It is important to highlight that when the analysis is conducted at the household level, important inequalities may be hidden. Therefore, contrary to expectations, in some countries the levels of multidimensional poverty of persons with and without disabilities are not significantly different, or households with members with disabilities can be better off than households without members with disabilities [9].

Various studies have been conducted to analyse the levels of multidimensional poverty of persons with disabilities around the globe. Some have analysed the levels of multidimensional poverty in children and compared the incidence of poverty in children with and without disabilities [10, 11]; other studies have computed the incidence of multidimensional poverty for persons with and without disabilities using individual data [5, 7, 12], and others computed a multidimensional poverty index for persons with disabilities [13]. Most studies have used national household surveys, which provide information on the incidence and intensity of multidimensional poverty at the national level for rural and urban areas, and in some cases for regions. However, in most cases it is not possible to analyse the levels of multidimensional poverty in municipalities or at the lowest level of disaggregation. In the case of disability statistics, there are only a few studies including Mont et al. [14] analysing the prevalence of disability at the municipality level, and in most cases it is not possible to disaggregate by type of impairment.

In Colombia, a few studies have analysed the levels of multidimensional poverty for persons with disabilities and compared those with the levels experienced by persons without disabilities [6]. In most studies, persons with disabilities are more likely to be multidimensionally poor and face a higher number of deprivations [9]. In addition, persons with disabilities in Colombia face a range of barriers to their access to basic services and opportunities; for example, children with disabilities have lower levels of school attendance compared with children without disabilities, and the percentage of women with disabilities who are outside the labour force is much higher than it is for women without disabilities [2].

The Colombia multidimensional poverty index (MPI), which was officially launched in 2010, has five dimensions and 15 indicators [15]. Since 2010 it has been the official measure of multidimensional poverty in the country, and, given its relevance, it has also been computed using the national household and population Census of 2005 and 2018. The national MPI has been used to analyse the levels of poverty in the different municipalities, and it is the only poverty measure that is computed at that level of disaggregation [16].

Although Colombia the number of studies analysing data on disability has increased [2], there is no clear analysis of their levels of multidimensional poverty, and how those differ from those of persons without disabilities. Most importantly, to date, there has been no clear analysis of the incidence of multidimensional poverty for persons with disabilities at the municipal level, and no evidence of a disability development gap within the country. The purpose of this article is therefore to analyse the levels of multidimensional poverty experienced by persons with disabilities and to disaggregate the data by type of impairment. We compared the incidence and intensity of multidimensional poverty experienced by persons with and without disabilities, and we analysed the differences in the levels of deprivation by indicator at the municipality level.

## 2 Methodology

### 2.1 Data

We used the Colombian National Household and Population Census from 2018. The census collected information for all individuals living in households and institutions in the country. It used two data collection methods: online questionnaires and face-to-face interviews. Data were collected from January to October 2018 and included information on household and individual characteristics, education, health, employment, migration, ethnicity, disability, and living standards. The census collected data in the 32 departments, 1,101 municipalities, 20 areas not in municipalities, and in San Andres, Providence, and Santa Catalina Island. It collected data from 48,258,494 individuals in the country and it is the only source of information that can be disaggregated at the municipality level [17]. The researchers had access to the census data after requested access to the annonimized microdata to DANE and only have access to the data through computers from the statistics office. To create the maps presented in this article, we used the shapefiles created by the National Statistics Office, which are publicaly avaiable [18].

In addition to the census, we used the C600 register, which includes information from all the schools and educational institutions (official and unofficial) that provide services from kindergarten to high school, or other types of educational service in rural and urban areas of Colombia. The final sample of education institutions was 53.066 [19].

### 2.2 Disability definition

The census included questions on disability suggested by the Washington Group on Disability Statistics [20]. However, the short set of questions was presented only to individuals who reported living with a disability in answer to this question: Given your physical and mental condition, and without any help or support, in your daily living do you have difficulties doing activities such as: hearing, speaking, seeing, moving your body, walking, holding objects with your hands, understanding, learning, remembering, eating, getting dressed or interacting with others? If the person answered yes, she or he was presented with a list of nine activities and a four-level severity scale (cannot do it/ with severe difficulty/ with some difficulty/ no difficulty).

In this study we defined a person with disability as one who reported having severe difficulty or inability to perform at least one of the nine activities (hearing, speaking, seeing, moving or walking, grasping objects, understanding, eating or dressing or bathing oneself, interacting with others, or any other activity without presenting symptoms of heart disease). We also computed a second disability variable, which categorised a person with disability if the person reported having some difficulty or severe difficulty or inability to do at least one of the activities, and we conducted a different analysis using this definition; the results are available on request.

### 2.3 Methods

The Colombian Multidimensional Poverty Index (MPI) uses the Alkire-Foster (AF) method [21, 22]. The AF method allows for the selection of binary deprivation/non-deprivation indicators, their relative weights, the specification of deprivation cut-offs (i.e., the point at which a certain deprivation/non-deprivation applies), and overall multidimensional cut-offs. Because of this, the AF method is known as a dual cut-off method. The AF method creates a deprivation profile for each unit of identification (e.g., households). Based on specific deprivation cut-offs, a household and all its members are first identified as either deprived or non-deprived in each indicator. These binary deprivation indicators are then multiplied by the respective weights that have been selected for each indicator. These weights reflect the importance of each indicator for overall socio-economic deprivation. Each weighted deprivation profile is then summarised as an overall deprivation score and compared against one or more overall multidimensional cut-offs.

The aggregate-level measures most commonly constructed with the AF method are the so-called adjusted headcount ratio or *M*_0_, and its two partial indices *H* and *A*. *H* is the headcount ratio or incidence of multidimensional poverty. It expresses the percentage of people who are multidimensionally poor, given the chosen multidimensional cut-off. *A* is the intensity and expresses the average share of weighted deprivations faced by multidimensionally poor individuals. The *M*_0_ is an adjusted headcount ratio, which is the result of multiplying the incidence by the intensity (*H* × *A*). It represents the deprivations experienced by persons who are multidimensionally poor (0–1), expressed as a percentage of the total possible deprivations, i.e., the deprivations that would be experienced if everyone in society was deprived in all indicators included in the measure in question [22]. In additional to the *H*, *A* and *M*_0_ or *MPI*, using the AF method it is possible to compute the uncensored headcount ratios, or the percentage of individuals deprived in each indicator, also the censored headcount ratios, or the percentage of people who are deprived in an indicator AND multidimensionally poor, and the absolute and percentage contributions of each indicator to the *MPI* (for more details, please see Alkire et al. [22]. It is important to highlight that there is a relationship between the censored headcount ratios and the *MPI*, given that the *MPI* can also be computed as the weighted sum of the censored headcount ratios.

We computed the structure of the Colombian MPI, following Angulo-Salazar et al. [15]. The Colombian MPI has five dimensions and 15 indicators. The five dimensions (indicators) are health (health insurance and access to health services), education (educational achievement, literacy), employment (dependency, informal employment), child and youth conditions (school attendance, school lag, access to child-care services, and child labour), and service and housing conditions (water, sanitation, floor and wall materials, and overcrowding). All are computed at the household level and assume that household deprivations and achievements are experienced by all household members (Table 61 in S1 Appendix). The Colombia MPI uses nested weights, and therefore all five dimensions are equally weighted, and indicators inside each dimension share the same relative weight. The poverty line was defined at 33 per cent; thus a household and all its members are considered multidimensionally poor if they face 33 per cent or more of the weighted sum of deprivations. The poverty line represents the minimum level of deprivations that a household needs to face in order to be considered multidimensionally poor. The normative and statistical arguments explaining why 33 per cent was selected can be found in Angulo-Salazar et al. [15].

The National Household and Population Census (2018) included information to compute nine of the 15 indicators included in the MPI. As with the 2005 census, the indicator on informality was replaced by an indicator capturing deprivations related to dependency. All the other five indicators were computed using information from administrative records, which were merged with the census data by the National Statistics Office of Colombia (DANE) and were available to the researcher on request.

Using the information provided by the national MPI for Colombia, we disaggregated the data of the MPI by disability status of the household, analysing the levels of multidimensional poverty of households with and without members with disabilities. In addition, we computed a double disaggregation (by municipality and disability status), aiming to compare the levels of multidimensional poverty of households with and without members with disabilities in all the municipalities of the country. We compared the incidence, intensity, MPI, and uncensored and censored headcount ratios between households with and without members with disabilities. Finally, using the information from the C600 register, we triangulated the results of the national MPI, especially those related to deprivations in school attendance, with the number of teachers and schools that reported children with disabilities on their registers. Using this information, we analysed the distribution of deprivations and schools in the country, and the association between both indicators.

## 3 Results

Table 1 presents the main characteristics of persons with disabilities in Colombia. Using the main definition of disability, 4.1 per cent of the population in Colombia live with some type of functional difficulty; of those, 54 per cent are women and 46 per cent are men. In addition, 73 per cent live in urban areas, and 27 per cent in rural areas—a percentage that is significantly lower than the percentage of persons without disabilities that live in urban areas in the country. On average, persons with disabilities have 3.7 years of education, compared with the 5.4 years of education typical of persons without disabilities. As expected, a larger percentage of the population with disabilities is aged 60 years or older (41.4 per cent). Finally, 45.4 per cent of persons with disabilities are heads of households (Table 1).

**Table 1:** Descriptive statistics.

Variables	All	WD: Yes	WD: No	Difference
	(1)	(2)	(3)	(4)
A. Statistics for individuals				
Men (%)	48.63	45.99	48.83	-2.84***
	(49.98)	(49.84)	(49.99)	
Women (%)	51.37	54.01	51.17	2.84***
	(49.98)	(49.84)	(49.99)	
Urban (%)	77.35	73.01	77.68	-4.68***
	(41.86)	(44.39)	(41.64)	
Rural (%)	22.65	26.99	22.32	4.68***
	(41.86)	(44.39)	(41.64)	
Head of Household -HH- (%)	32.49	45.36	31.50	13.86***
	(46.83)	(49.78)	(46.45)	
Years of schooling	5.41	3.75	5.55	-1.80***
	(3.72)	(3.62)	(3.69)	
Age [0,15)	22.66	8.51	23.75	-15.23***
	(41.86)	(27.91)	(42.55)	
Age [15,30)	25.86	12.69	26.87	-14.18***
	(43.79)	(33.29)	(44.33)	
Age [30,45)	21.10	13.37	21.69	-8.32***
	(40.80)	(34.03)	(41.21)	
Age [45,60)	17.10	24.04	16.57	7.47***
	(37.65)	(42.74)	(37.18)	
Age (>60)	13.28	41.38	11.12	30.26***
	(33.94)	(49.25)	(31.44)	
N (%)	100	7.13	92.87	
B. Statistics for households				
Men HH (%)	59.26	54.69	59.77	-5.08***
	(49.13)	(49.78)	(49.04)	
Women HH (%)	40.74	45.31	40.23	5.08***
	(49.13)	(49.78)	(49.04)	
Years of schooling of the HH	5.64	3.78	5.86	-2.08***
	(3.82)	(3.64)	(3.78)	
Age of the HH	47.64	60.53	46.21	14.32***
	(16.54)	(16.37)	(15.93)	
Household size	2.04	1.96	2.05	-0.09***
	(1.27)	(1.26)	(1.28)	
# of children in the household	0.60	0.37	0.62	-0.25***
	(0.89)	(0.74)	(0.90)	
# of elderly residents in the household	0.41	0.81	0.36	0.45***
	(0.69)	(0.81)	(0.66)	
N (%)	100	13.59	86.41	

*Note*: Own calculations using National Population and Household Census data 2018. WD: With Disabilities. In parenthesis is the standard deviation. The first column is the estimate for the whole population. The second column is the estimate for persons and households with at least one member with disabilities. The third column is the estimate for persons and households without members with disabilities. The fourth column is the difference between column 2 and 3.

***p<0.01;

**p<0.05;

*p<0.1

When we analysed the characteristics of households with members with disabilities, we found that, on average, the heads of households that included members with disabilities had, on average, fewer years of education than the heads of households without members with disabilities. In addition, these households have on average a larger number of members older than 65 years, and lower numbers of children younger than 12 years old, compared with households without children with disabilities.

The results of the MPI reveal that persons with disabilities and their households are more likely to be multidimensionally poor. Indeed, 30.7 per cent of the households with at least one member with disabilities are multidimensionally poor (incidence), with an average intensity of 41.9 per cent, compared with 40.5 per cent of average deprivations for households without members with disabilities. When we analysed the levels of multidimensional poverty of households whose head had a disability, we found that these households are on average poorer than households whose head does not have disabilities. In addition, as expected, households in rural areas are poorer than households in urban areas, and this difference is maintained for households with members with disabilities (Table 2).

**Table 2:** Multidimensional poverty analysis.

	All	WD: Yes	WD: No	Difference
	(1)	(2)	(3)	(4)
A. National				
Incidence (H) (%)	17.22	30.70	15.10	15.60***
Intensity (A) (%)	40.77	41.90	40.59	1.31***
MPI	0.072	0.131	0.063	0.068***
B. Head of Household				
Incidence (H) (%)	17.22	28.99	19.41	9.59***
Intensity (A) (%)	40.77	42.42	42.37	0.04***
MPI	0.072	0.123	0.082	0.040***
C. Urban				
Incidence (H) (%)	13.75	19.70	13.15	6.55***
Intensity (A) (%)	41.15	41.71	41.07	0.64***
MPI	0.062	0.082	0.054	0.028***
D. Rural				
Incidence (H) (%)	43.99	52.22	42.76	9.46***
Intensity (A) (%)	43.75	43.09	43.87	-0.78***
MPI	0.193	0.225	0.187	0.037***

*Note*: Own calculations using Census data. WD: With Disabilities. The first column is the estimate for the whole population. The second column is the estimate for persons and households with disabilities. The third column is the estimate for persons and households without disabilities. The fourth column is the difference between column 2 and 3.

***p<0.01;

**p<0.05;

*p<0.1

When we disaggregated the data by type of limitation, we found important differences in the incidence of multidimensional poverty between types of difficulty. Persons living with difficulties involving understanding, eating, and relating with others face the greatest levels of multidimensional poverty, and households with members who have difficulties breathing have the lowest incidence of multidimensional poverty. For all groups the intensity of multidimensional poverty was higher than 40 per cent but lower than 50 per cent (Table 3).

**Table 3:** Incidence, intensity, and mpi by type of disability.

	H (%)	A(%)	MPI
Difficulty hearing voices or sounds	67.04	47.91	0.321
Difficulty speaking	74.69	49.13	0.367
Difficulty seeing near, far, or all around	61.24	46.52	0.285
Difficulty moving the body, walking, or going up and down stairs	71.89	48.54	0.349
Difficulty grasping or moving objects with the hand	67.69	47.02	0.318
Difficulty understanding, learning, or remembering or making decisions for themselves	74.94	48.95	0.367
Difficulty eating, dressing, or bathing themselves	78.69	51.46	0.405
Difficulty relating to or interacting with other people	74.42	46.72	0.348
Breathing difficulties	13.50	43.29	0.585
Multiple difficulties	30.43	42.65	0.129

*Note*: Multiple disabilities refer to households that include a household member with more than one type of disability.

**Fig 1:**
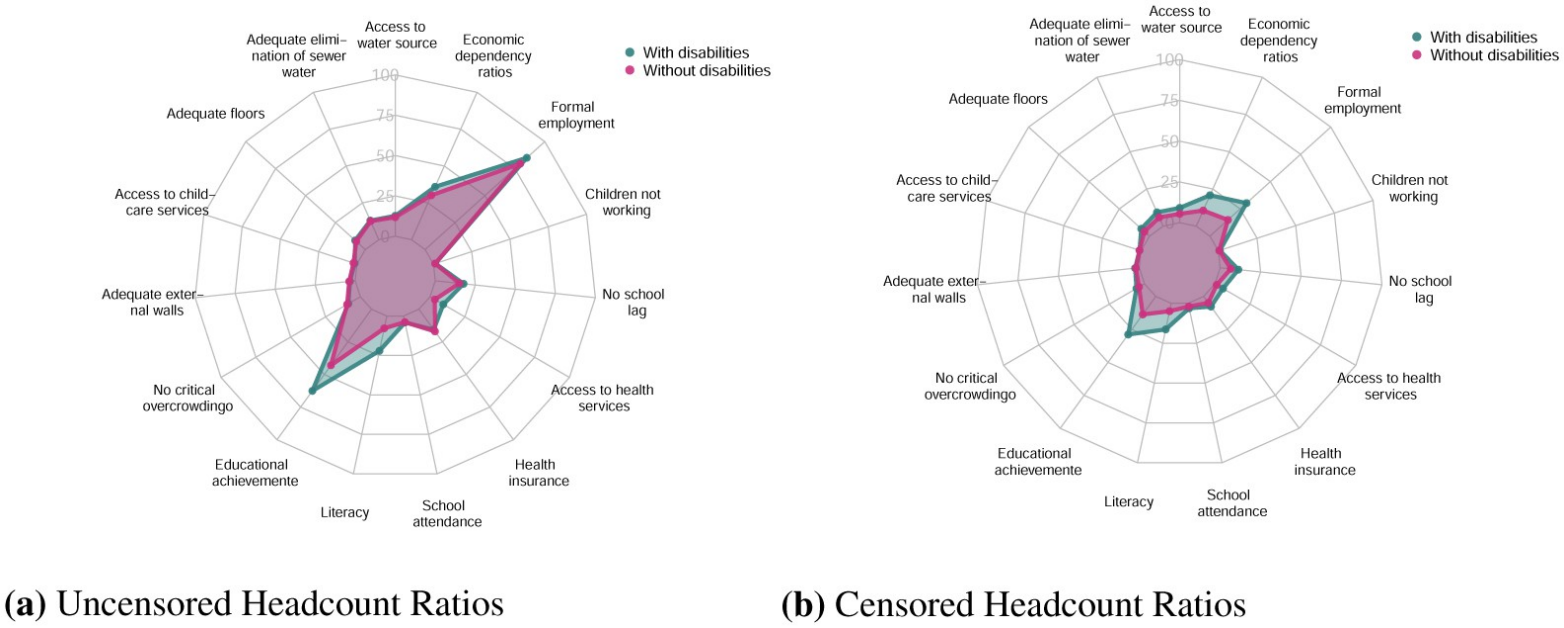
Uncensored and censored headcount ratios by disability status of the household. (**a**) Uncensored Headcount Ratios. (**b**) Censored Headcount Ratios.

The analysis of the uncensored headcount ratios (percentage of people who are deprived in each indicator) and the censored headcount ratios (percentage of people who are deprived and multidimensionally poor in each indicator) showed that persons with disabilities present higher levels of deprivation in education and employment indicators. These differences are larger when we analyse the censored headcount ratios of indicators such as Formal Employment and Economic Dependency Ratios (Fig 1).

Finally, when we analyse the percentage of people who are multidimensionally poor, but we use the definition of disability with three levels of severity, we find that households with members with mild and severe limitations have a higher incidence of multidimensional poverty, in comparison with households who do not have any member with a disability (Figure 61 in S1 Appendix). In addition, the severity of the disability of the household member is a factor associated with higher incidence and intensity of poverty. Indeed, 33.8 per cent households with at least one household member with severe limitation in at least one of the domains are multidimensionally poor, in comparison with households whose member has a mild disability (25.8 per cent). Regarding the intensity of the disability, something similar is observed: the higher the severity of the functional difficulty, the higher is the intensity of their poverty on average. A similar pattern is observed when we analyse the levels of deprivation of each subgroup: households with members with severe disability are the ones with the largest uncensored and censored headcount ratios in most of the indicators, except in child labour, access to health-care services, access to child care, and health insurance. In this last indicator, households with members with severe disabilities present lower uncensored headcount ratios, with larger censored headcount ratios, in comparisons with household without members with disabilities, or with members with less severe disability; therefore, a larger percentage of households with members with severe limitations are deprived in this indicator and are multidimensionally poor (Table 4).

**Table 4:** Incidence (H), intensity (A) and Multidimensional Poverty Index (MPI) by type of disability.

	WD: No		WD: Yes—Mild		WD: Yes—Severe	
	(1)	(2)	(3)	(4)	(5)	(6)
	UHR	CHR	UHR	CHR	UHR	CHR
N (%)	86.41		5.30		8.29	
H (%)	15.1		25.80		33.82	
A (%)	40.59		41.41		42.13	
MPI	0.063		0.109		0.144	
Access to a Clean Water Source	11.76	5.42	12.40	8.29	12.80	9.55
Sanitation	12.57	5.95	12.66	8.43	13.20	9.84
Floor Materials	7.57	4.22	7.96	6.03	8.79	7.26
Wall Materials	3.74	2.11	3.06	2.25	3.42	2.76
Overcrowding	9.50	3.90	8.37	5.09	8.83	6.05
Educational Achievement	42.99	13.65	55.54	23.89	67.04	32.05
Literacy	7.78	4.91	14.51	11.15	26.85	19.67
School Attendance	3.87	1.98	3.22	2.42	4.69	3.50
School Lag	15.14	6.61	17.56	10.33	17.78	11.80
Child Labour	1.10	0.57	1.25	0.94	1.18	0.93
Access to Health Services	3.43	1.27	10.61	5.90	8.79	5.63
Dependency	30.13	10.53	32.03	16.89	38.39	23.26
Informal Employment	79.52	14.88	83.63	25.48	85.92	33.33
Access to Child-care Services	2.27	0.98	1.49	0.93	1.49	0.99
Health Insurance	17.02	4.88	15.95	6.89	15.47	8.22

*Note*: Own calculations using Census data. WD: With Disabilities; UHR: Uncensored Headcount Ratio; CHR: Censored Headcount Ratio. The first column is the UHR estimate for households without members with disabilities. The second column is the CHR estimate for households with disabled members. The third column is the UHR estimate for households with members with mild disability. The fourth column is the CHR estimate for households with members with mild disability. The fifth column is the UHR estimate for households with members with severe disabilities. The sixth column is the CHR estimate for households with members with severe disabilities. Multiple disabilities refer to households that include an individual with more than one type of disability.

### 3.1 Results by municipalities

**Fig 2:**
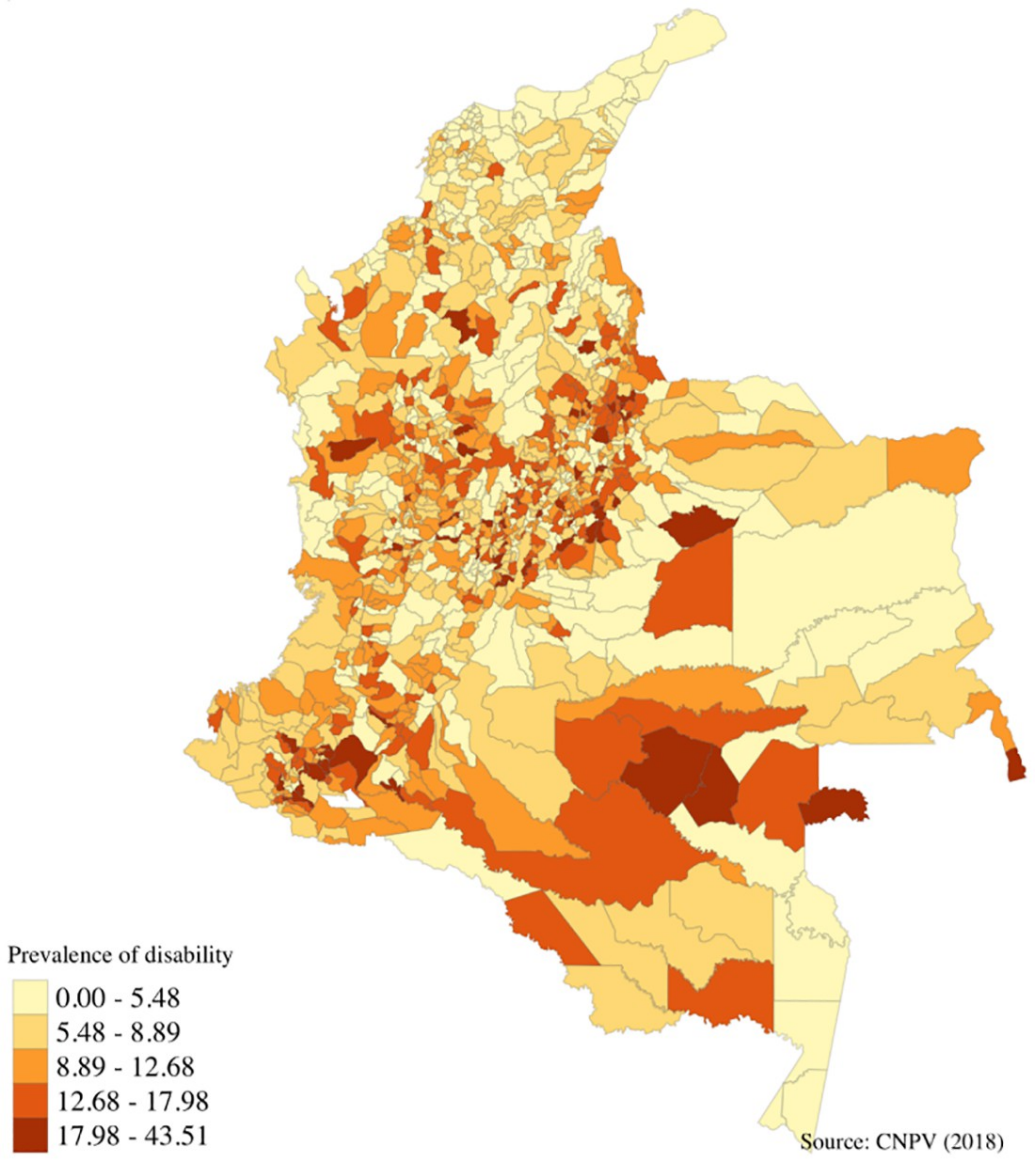
Prevalence of population with disabilities by municipality. Source: Authors own calculations using Census 2018 and shapefiles from DANE [19].

The prevalence of disability varies between municipalities, as can be seen in Fig 2. Only in six of the municipalities did we find that households without members with disabilities have higher levels of multidimensional poverty. In the case of Papunahau in Vaupez, the incidence of multidimensional poverty was 5 percentage points (pp) higher than for households with members with disabilities. By contrast, in 62 per cent of municipalities the difference between the incidence of multidimensional poverty for households with and without members with disabilities is larger than 20 pp.

**Fig 3:**
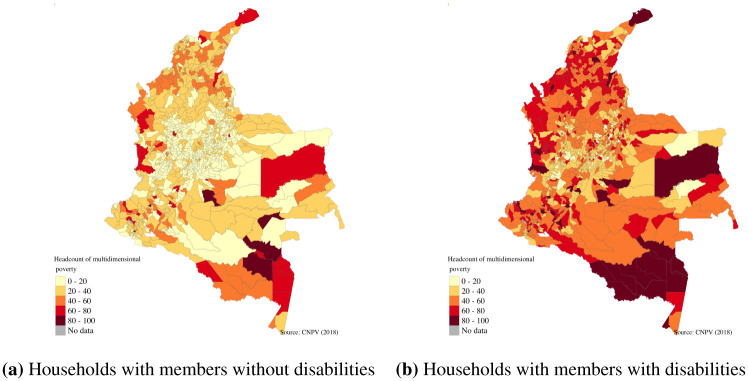
Incidence of multidimensional poverty for households with and without members with disabilities in Colombia. (**a**) Households with members without disabilities. (**b**) Households with members with disabilities. Source: Authors own calculations using Census 2018 and shapefiles from DANE [19].

In addition, only in 57 municipalities (less than 5 per cent of the total municipalities) were the levels of multidimensional poverty typical of households with members with disabilities lower than the national average. On the contrary, in 524 municipalities households without members with disabilities have lower levels of multidimensional poverty compared with the national mean (Fig 3).

**Fig 4:**
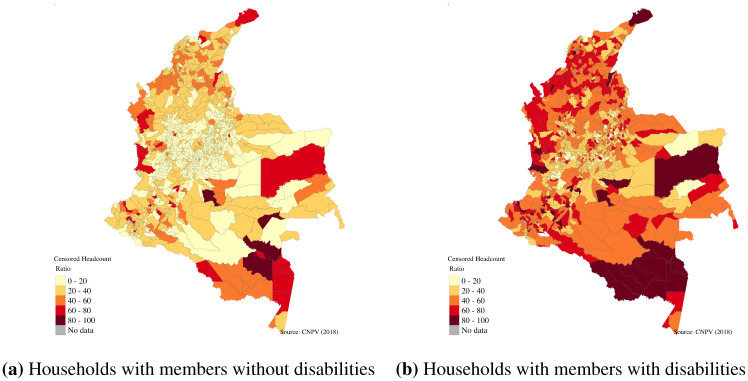
Deprivation in informal employment for households with and without members with disabilities in Colombia. (**a**) Households with members without disabilities. (**b**) Households with members with disabilities. Source: Authors own calculations using Census 2018 and shapefiles from DANE [18].

**Fig 5:**
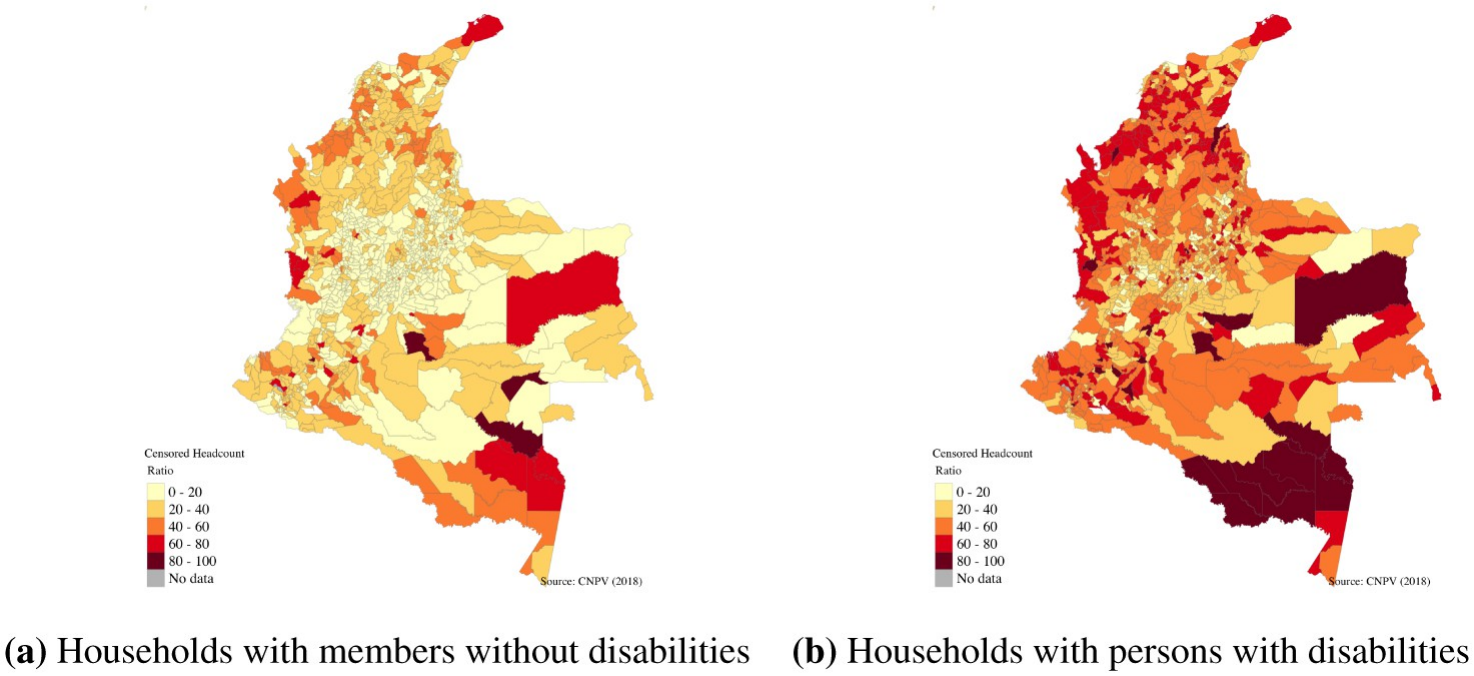
Deprivation in years of schooling for households with and without members with disabilities by municipality in Colombia. (**a**) Households with members without disabilities. (**b**) Households with persons with disabilities. Source: Authors own calculations using Census 2018 and shapefiles from DANE [18].

The mapping of the levels of multidimensional poverty of households with and without members with disability at the municipality level revealed important inequalities. For example, households with members with disabilities in all municipalities of the country presented higher levels of multidimensional poverty, and intensity. Fig 3 reveals that even in regions where the levels of multidimensional poverty are low (for example, Bogota, capital city of the country), households with members with disabilities are on average poorer than households without members with disabilities. At the indicator level, the differences observed between households with and without members with disabilities are larger in indicators such as education and employment, as was observed at the national level (Figs 4 and 5). As with the incidence, the intensity and multidimensional poverty levels of households with members with disabilities were much higher compared with households without disabilities.

We also analysed the levels of deprivation for households with and without members with disabilities in different indicators. As presented in Fig 1, households with members with disabilities present higher levels of deprivation in employment and education indicators. When we disaggregated those indicators and analysed the differences between households with and without members with disabilities, we found that in all municipalities of the country persons with disabilities face higher levels of deprivation in terms of informal employment (Fig 4) and also have higher levels of deprivation in terms of years of schooling (Fig 5), and therefore a larger number of households with members with disabilities have members working in informal employment or without nine years of education.

**Fig 6:**
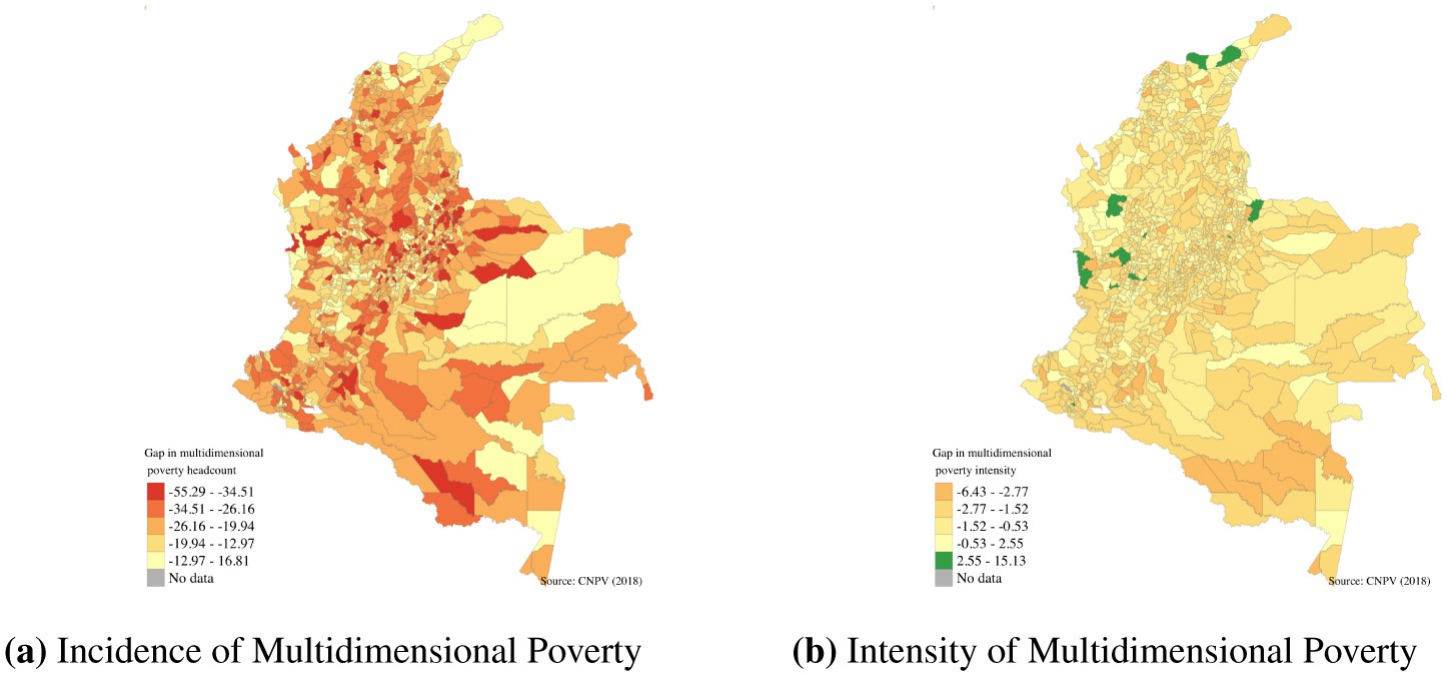
Gap between households with and without members with disabilities by municipality in Colombia. (**a**) Incidence of Multidimensional Poverty. (**b**) Intensity of Multidimensional Poverty. Source: Authors own calculations using Census 2018 and shapefiles from DANE [18].

When we analysed the gap in the incidence and intensity of multidimensional poverty of households with and without members with disabilities in each municipality, we found that households with members with disabilities present a higher incidence, but in some municipalities the intensity of their poverty is lower (Fig 6). In addition, when we analysed the gap in the incidence of multidimensional poverty and how it was related to the prevalence of disability, we found that municipalities with a larger prevalence of disability present a higher gap (Fig 62 in S1 Appendix).

### 3.2 Deprivations in education and provision of education services

**Fig 7:**
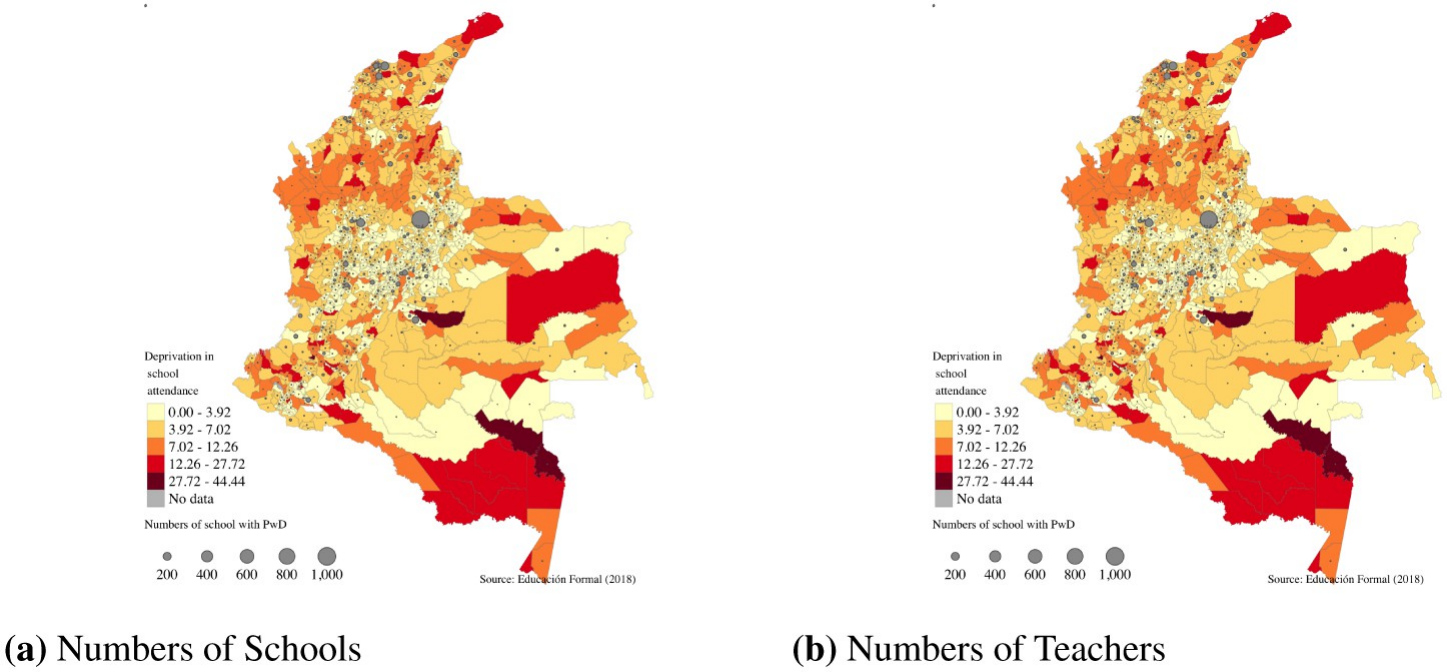
Numbers of schools and teachers for people with disabilities. (**a**) Numbers of Schools. (**b**) Numbers of Teachers. Source: Authors own calculations using Census 2018 and shapefiles from DANE [18] alMGN.

Aiming to analyse in more detail some of the main reasons associated with the differences in the levels of deprivation on indicators related to education, we triangulated information on the levels of deprivation in school attendance and the number of inclusive schools and specialist teachers for children with disabilities. We found that most schools that include children with disabilities are in the main cities, with an unequal distribution of schools and teachers across the country. In addition, municipalities with the highest deprivation in terms of school attendance are the ones with the lowest number of schools and teachers for children with disabilities in the country (Fig 7).

## 4 Discussion

Persons with disabilities around the globe constitute a socially excluded group. They usually face higher levels of poverty and deprivation, and their levels of achievement in various aspects of life are limited [23]. This study aimed to analyse the differences between the levels of multidimensional poverty and deprivation of households with and without members with disabilities in Colombia at the national and municipality levels, using the National Population and Household Census. The results revealed that households with members with disabilities are on average poorer than households without members with disabilities. They usually face higher levels of deprivation, and the intensity of their poverty is higher compared with that of households without members with disabilities. In addition, important differences are observed at the municipality level, where households with members with disabilities usually face higher levels of deprivation on indicators related to education, health, and employment.

Disability and poverty are two related conditions, with disability increasing the risk of poverty, and poverty increasing the risk of disability [3]. In the context of Colombia, there are important inequalities between the levels of poverty and deprivation of persons with and without disability, but also for individuals with different types of functional limitation. In Colombia, according to the research results, persons with functional limitations in eating, dressing or bathing themselves are the group that presents the highest incidence and intensity of multidimensional poverty. This finding confirmed the ones reported by [24], where the authors found that persons with self-care limitations were the second poorest group among persons with disabilities.

Although Colombia signed and ratified the Convention on the Rights of Persons with Disabilities in 2011, and has implemented various policies to guarantee the rights of persons with disabilities [24], the results of this paper reveal that there are important gaps in the access to services available to persons with disabilities in Colombia. For example, a higher percentage of persons with disabilities live in households with members who have lower levels of education. Although it was not possible to analyse whether the member of the household who was creating the deprivation was living with disability, it is possible to conclude that persons with disabilities and their families face lower opportunities, and therefore their levels of social and economic development are lower. Also, when we correlated the number of schools providing inclusive services for children with disabilities and the levels of deprivation in school attendance, we found that the poorest municipalities are the ones with lower numbers of schools specialising in the needs of children with disabilities, a fact which increases the children’s risk of poverty, reduces their accumulation of human capital, and reduces their probability of overcoming poverty.

The findings of this article support previous findings analysing the levels of multidimensional poverty experienced by persons with disabilities in a range of Latin American countries, including Colombia [6, 9]. Although it has been recognised that persons with disabilities in the region and the country present higher levels of deprivation and poverty [2], little information can be found on the type of social protection policies implemented by the country to reduce its levels of poverty [25] and to support the extra needs of individuals with dis abilities and their families. Indeed, an analysis of the social protection system in the country revealed that there are no exclusive benefits for persons with disabilities, and only adults older than 60 who have a certificate of disability are prioritised for a social pension [25]. In addition, conditional cash transfers, whose main objective is to reduce inter-generational poverty and improve human capital, have included children with disabilities, but there is no evidence of a positive effect on their educational achievement [26].

In this context, it is necessary that countries such as Colombia implement policies aiming to reduce the poverty and deprivation of persons with disabilities and recognise their additional needs. These policies should be comprehensive and guarantee access to services and basic opportunities such as education and employment. In addition, implementing inclusive social protection systems will protect persons with disabilities from the extra costs of living with a disability and will support their access to education, health, and employment.

Finally, it is important to emphasise that the MPI can be used as a tool for policy. Thus disaggregating it by disability status provides important information to policy makers for the design and implementation of policies. Therefore, it is necessary to analyse the levels of deprivation of persons with disabilities and recognise their needs, and to ensure that this information contributes to the inclusive implementation of policies to reduce poverty.

### 4.1 Limitations

Although the results of this study reveal important inequalities between households with and without members with disabilities, there are some limitations that should be considered. First, given the filter question included in the Census, it is possible that some individuals who present functional difficulties have not been captured. In addition, there are important differences in the prevalence of disability by municipality, an aspect that may be associated with the type of question included in the Census. Also, the fact that the measure of multidimensional poverty is computed at the household level, limits the analysis of intra-household inequalities and might conceal the likelihood that persons with disability face higher deprivations in comparison with other household members.

## 5 Conclusions

Persons with disabilities and their families face higher levels of multidimensional poverty and deprivation compared with persons without disabilities, and when the data are analysed for all municipalities, the results reveal that in Colombia persons with disabilities and their families are poorer than households without members with disabilities. In addition, these households face higher levels of deprivation in indicators related to education, employment, and health. Finally, the provision of educational services for children with disabilities in the country is limited in municipalities with higher percentages of households with members with disabilities who are also deprived in terms of school attendance, a fact which reveals an important lack of service provision for persons with disabilities in the country.

## Supporting information

S1 Appendix (PDF)
