# Supplementary material for: Multidimensional poverty among persons with disabilities in Colombia: Inequalities in the distribution of deprivations at the municipality level
Source: PLoS One. 2023 Jun 29;18(6):e0286983. doi: 10.1371/journal.pone.0286983 (PMC10309615; doi:10.1371/journal.pone.0286983)

Appendix

Table A1. Dimensions, Indicators, Deprivation Cut-offs and Weights of the MPI

Dimensions	Variable	Indicator
Education	Educational Achievement (1/10)	A household is deprived if at least one person over 15 years of age is not attending school and does not have 9 years of education.
	Literacy (1/10)	A household is deprived if at least one person older than 15 years cannot read and write.
Childhood and Youth Conditions	School Attendance (1/20)	A household is deprived if at least one school-age child (aged 6 to 16 years) does not attend school.
	School Lag (1/20)	A household is deprived if at least one school-age child is one or more years behind the school year according to her age.
	Access to Child-care Services (1/20)	A household is deprived if at least one child aged 0-5 years simultaneously lacks access to health care, nutrition, and education.
	Child Labour (1/20)	A household is deprived if at least one child aged 5 to 17 years worked in the week previous to the survey.
Employment	Dependency (1/10)	A household is deprived if its economic dependency ratio is greater than or equal to 3.
	Informal Employment (1/10)	A household is deprived if at least one member currently in employment does not contribute to a pension scheme.
Health	Health Insurance (1/10)	A household is deprived if at least one member does not have health insurance.
	Access to Health Services (1/10)	A household is deprived if at least one household member did not have access to health care services when needed.
Services and Housing Conditions	Access to Water (1/25)	An urban household is deprived if it does not have a public aqueduct.
		A rural household is deprived if its drinking water is obtained from an open well, river, canal, stream, pond, tanker truck, bottle, or other non-plumbed source.
	Sanitation (1/25)	An urban household is deprived if it does not have access to a public sewerage system.
		A rural household is deprived if it has a non-connected toilet or latrine, or it does not have a toilet.
	Floor Materials (1/25)	A household is deprived if it has dirt floors.
	Wall Materials (1/25)	An urban household is deprived if the exterior walls are built of untreated wood, boards, planks, guadua or other vegetation, zinc, cloth, cardboard, or waste material, or if it does not have exterior walls.
		A rural household is deprived when exterior walls are built of guadua or other vegetation, zinc, cloth, cardboard, waste materials or if it does not have exterior walls.
	Overcrowding (1/25)	A household is deprived if 3 or more people share a room (excluding kitchen, bathroom, and garage).

Table A2. Incidence, Intensity, Multidimensional Poverty and Deprivations by Regions.

Regions / Variables	Bogota			Caribe			Centro Oriente			Centro Sur			Eje Cafetero y Antioquia			Llanos			Pacific		
	(1)	(2)	(3)	(4)	(5)	(6)	(7)	(8)	(9)	(10)	(11)	(12)	(13)	(14)	(15)	(16)	(17)	(18)	(19)	(20)	(21)
	WD: Yes	WD: No	Diff	WD: Yes	WD: No	Diff	WD: Yes	WD: No	Diff	WD: Yes	WD: No	Diff	WD: Yes	WD: No	Diff	WD: Yes	WD: No	Diff	WD: Yes	WD: No	Diff
H (%)	11.86	5.25	***	45.35	26.61	***	30.74	11.88	***	40.19	18.32	***	26.33	11.42	***	41.67	18.85	***	32.30	16.57	***
A (%)	40.62	39.06	**	43.92	42.80	**	41.25	39.78	**	42.10	40.59	**	41.44	40.16	**	43.22	41.55	**	41.85	40.57	**
MPI	0.048	0.025	***	0.201	0.117	***	0.128	0.048	***	0.171	0.075	***	0.112	0.046	***	0.183	0.082	***	0.138	0.069	***
Access to water	0.57	0.48		15.73	17.58	**	15.33	11.84	***	19.89	18.04	*	10.32	8.79	**	19.81	16.88		14.96	15.53	*
Sanitation	0.75	0.70		27.6	27.42		9.69	7.77	*	14.49	12.84	**	10.72	8.95	*	16.29	14.43		15.12	15.60	
Floor Materials	0.35	0.24		18.00	18.61		7.26	5.15	*	8.87	7.80	*	3.00	2.98		13.10	11.00		12.76	8.75	***
Walls Materials	0.41	0.30		6.04	7.53	**	2.12	1.97		4.87	4.87		2.54	2.52		8.36	7.56		3.68	4.52	*
Overcrowding	5.68	5.60		17.72	19.03	*	7.60	8.27		8.82	8.93		6.07	6.04		11.24	11.10		7.08	7.09	
Educational Achievement	39.94	24.09	***	63.45	47.14	***	67.15	45.54	***	74.49	53.43	***	64.28	43.70	***	71.83	50.52	***	66.25	46.67	***
Literacy	10.15	1.69	***	32.90	14.46	***	21.38	6.47	***	25.62	8.43	***	20.93	6.94	***	24.69	7.60	***	22.49	8.00	***
School Attendance	2.22	1.89		5.96	5.73		3.65	3.42		5.53	4.72	*	3.86	3.56		5.94	4.60		3.97	3.89	
School Lag	14.21	11.08	**	22.77	19.33	**	16.53	14.29	**	19.57	16.11	**	17.53	14.36	**	22.41	17.81	**	16.25	14.81	**
Child Labour	0.45	0.50		1.06	1.08		1.30	1.17		2.28	1.89	**	1.23	1.05		1.69	1.42		1.27	1.26	
Access to Health Services	9.47	3.51	***	10.77	3.62	***	9.30	3.48	***	11.76	3.84	***	7.41	2.54	***	13.73	4.89	***	9.31	3.56	***
Dependency	23.09	18.25	*	46.32	41.53	*	37.20	28.90	*	42.66	34.81	***	31.77	25.82	*	44.86	36.07	*	36.31	31.11	*
Informal Employment	74.57	68.30	**	91.77	88.37	**	86.32	79.39	**	90.74	85.24	**	81.12	74.79	**	89.45	82.91	**	87.32	82.54	**
Access to Child-care Services	1.28	1.82	***	1.90	2.95	***	1.37	2.09	***	1.53	2.25	**	1.23	1.79	***	2.40	3.12	***	1.53	2.46	***
Health Insurance	18.45	18.73	**	16.95	17.60	**	14.35	16.19	**	14.63	17.23	**	15.46	16.12	**	17.26	18.53	**	14.24	16.05	**

Note: Own calculations. WD Members with disabilities. The Bogota region is Bogota D.C. only. The Caribe region is the departments of Atlantico, Bolivar, Cesar, Cordoba, Sucre, Magdalena, La Guajira and San Andres Island. The Centro Oriente Region is the departments of Norte de Santander, Santander, Boyacá and Cundinamarca. The Centro Sur region is made up of the departments of Amazonas, Caquetá, Huila, Putumayo and Tolima. The Eje Cafetero and Antioquia Region are the departments of Caldas, Risaralda, Quindio and Antioquia. The Llanos Region includes the departments of Arauca, Casanare, Guanía, Guaviare, Meta, Vaupés and Vichada. The Pacific Region includes the departments of Chocó, Cauca, Nariño and Valle del Cauca. ***$p<0.01$; **$p<0.05$; *$p<0.1$

Fig A1. Uncensored and Censored Headcount Ratios for Households with Persons with Disabilities by Different Levels of Severity

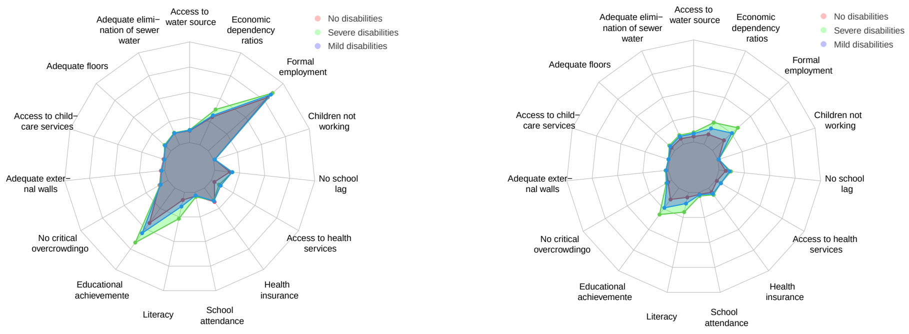

(a) Uncensored Headcount Ratio

(b) Censored Headcount Ratios

Fig A2. Gap of Multidimensional Poverty by Prevalence of Disability

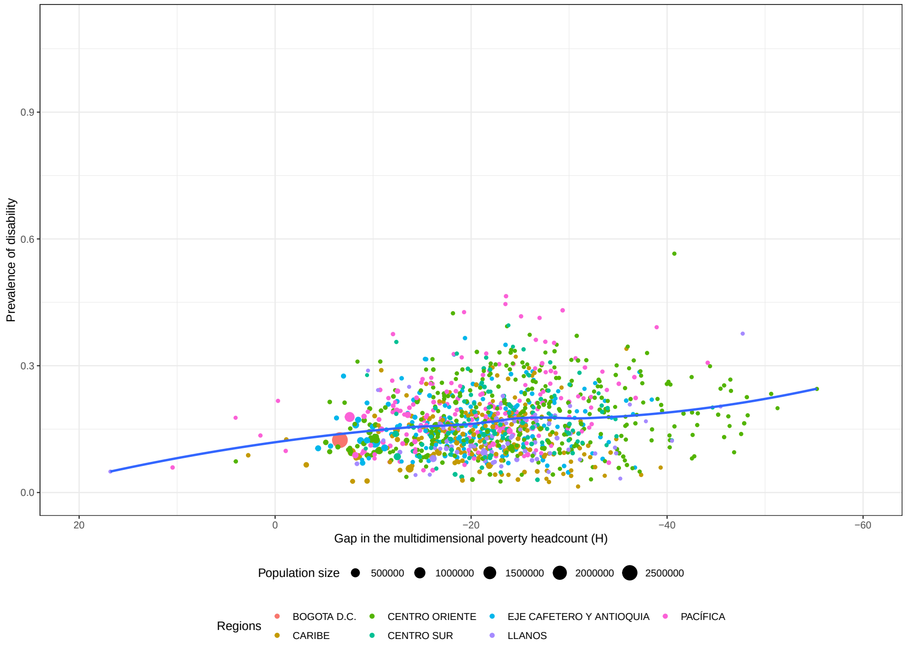

Supplement: S1 Appendix — (PDF) [file pone.0286983.s001.pdf]
